# Supplementary material for: The Novel Synthetic Antibiotic BDTL049 Based on a Dendritic System Induces Lipid Domain Formation while Escaping the Cell Envelope Stress Resistance Determinants
Source: Pharmaceutics. 2023 Jan 16;15(1):297. doi: 10.3390/pharmaceutics15010297 (PMC9866484; doi:10.3390/pharmaceutics15010297)
Supplement: Supplementary file 1 [file pharmaceutics-15-00297-s001.zip › pharmaceutics-2131715-supplementary.pdf]

pharmaceutics

Supporting Material

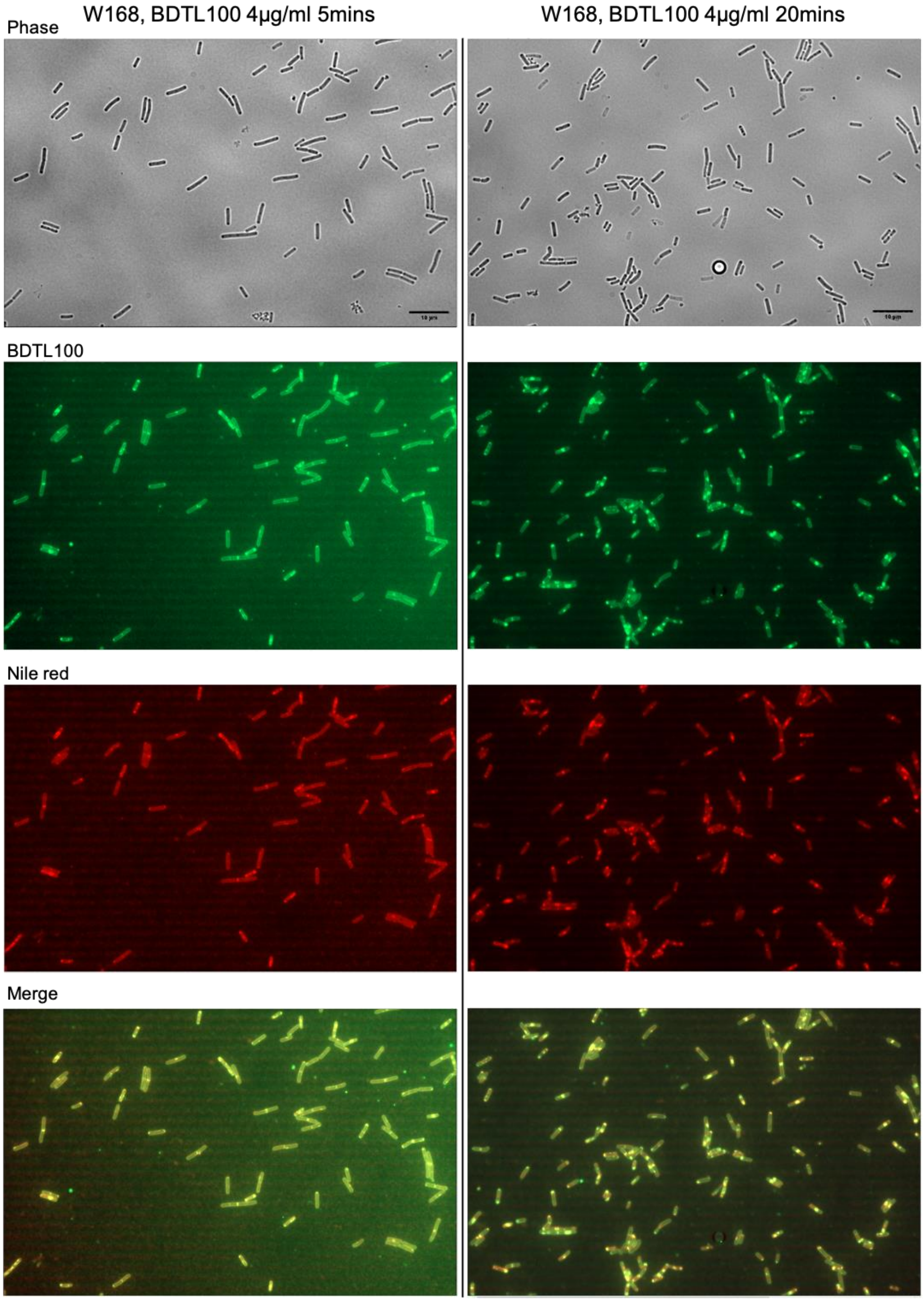

Supplement Figure S1. Effect of BDTL100 on *B. subtilis* membranes lipid packing state after 5 (left) and 20 (right) minutes, respectively. Top panels show phase contrast, followed by BDTL100 and Nile red fluorescence. Bottom shows merge. Scale bar is set to 10 μm.

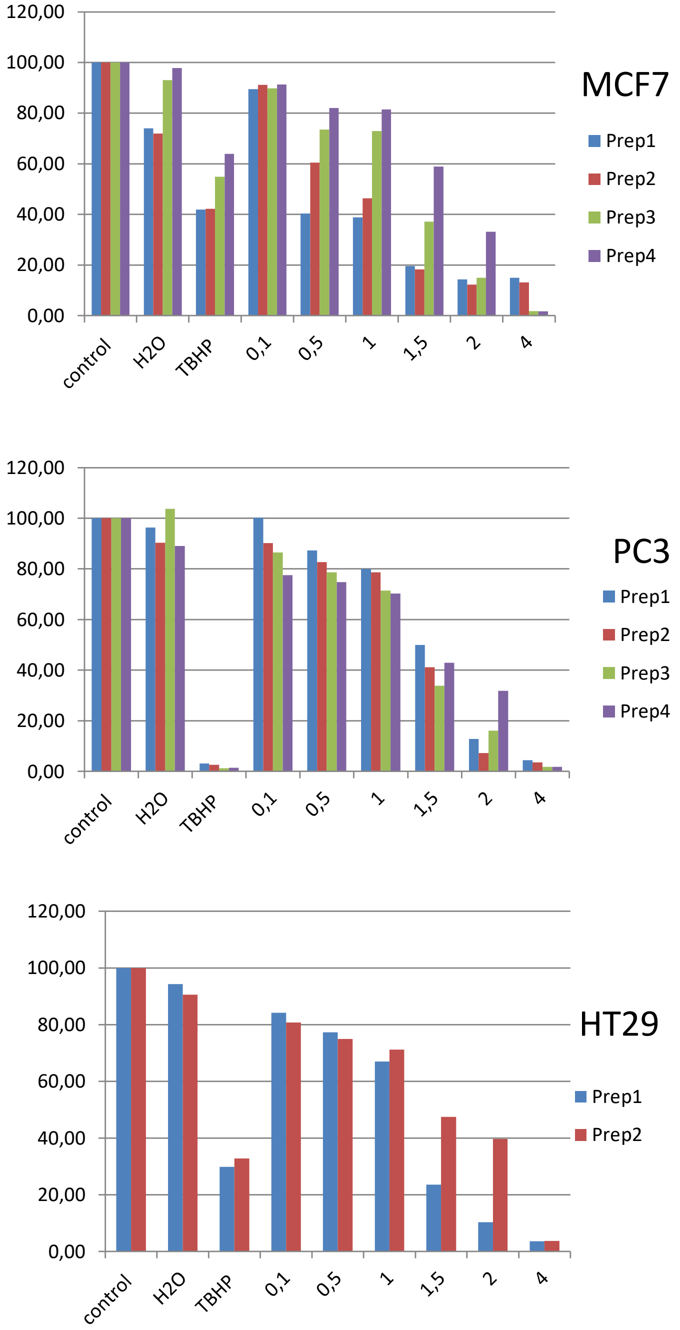

Supplement Figure S2. Cytotoxicity of BDTL049 at varrying concentrations against three different human cell lines *in vitro*. Data represent biological replicates.
